# Low-Cost Strain-Gauge Force-Sensing Sidestick for 6-DoF Flight Simulation: Design and Human-in-the-Loop Evaluation

**DOI:** 10.3390/s25144476

**Published:** 2025-07-18

**Authors:** Patrik Rožić, Milan Vrdoljak, Karolina Krajček Nikolić, Jurica Ivošević

**Affiliations:** 1 University of Zagreb, Faculty of Transport and Traffic Sciences, 10000 Zagreb, Croatia; patrik.rozic@fpz.unizg.hr (P.R.); jurica.ivosevic@fpz.unizg.hr (J.I.); 2 University of Zagreb, Faculty of Mechanical Engineering and Naval Architecture, 10000 Zagreb, Croatia; milan.vrdoljak@fsb.unizg.hr

**Keywords:** force-sensing joystick, flight simulator, fly-by-wire (FBW), human-in-the-loop (HITL), pitch-angle tracking, strain gauge, sensor calibration, human factors

## Abstract

Modern fly-by-wire (FBW) aircraft demand high-fidelity simulation systems for research and training, yet existing force-sensing solutions are often prohibitively expensive. This study presents the design, development, and validation of a low-cost, reconfigurable force-sensing sidestick. The system utilizes four strain-gauge load cells to capture pure pilot force inputs, integrated with a 6-DoF non-linear flight model. To evaluate its performance, a pitch-angle tracking task was conducted with 16 participants (pilots and non-pilots). Objective metrics revealed that the control strategy was a primary determinant of performance. Participants employing a proactive feedforward control strategy exhibited roughly an order of magnitude lower tracking-error variance than those relying on reactive corrections. Subjective assessments using the Cooper-Harper scale and NASA-TLX corroborated the objective data, confirming the sidestick’s ability to differentiate control techniques. This work demonstrates an open-source platform that makes high-fidelity FBW simulation accessible for academic research, pilot training, and human factors analysis at a fraction of the cost of commercial systems.

## 1. Introduction

The evolution of flight control systems from mechanical linkages to digital fly-by-wire (FBW) architectures represents a fundamental shift in aviation. This transformation, pioneered by NASA in the 1970s, has enhanced aircraft performance, safety, and efficiency by replacing heavy mechanical components with electronic signals [1,2]. In FBW systems, pilot inputs are interpreted not as direct commands for surface deflection but as targets for aircraft state, such as a desired pitch or roll rate, which are achieved through complex control laws. Consequently, the pilot’s control inceptor, typically a sidestick, often operates on force-sensing principles rather than large physical displacements. The design and characteristics of these inceptors have a significant impact on the pilot–aircraft system performance and handling qualities [3]. Operational experience with the first-generation F-16 sidestick showed that a completely rigid force-sensing design was “too sensitive”, prompting engineers to add roughly 6 mm of artificial travel to improve feel and reduce cross-axis coupling [4]. Equivalency testing by Rummel [5] showed that pilots tolerate a pure force-sensing stick but significantly prefer a hybrid design with a few millimeters of travel, highlighting the importance of tactile cues. This paradigm shift has intensified the demand for high-fidelity flight simulators that can accurately reproduce the tactile cues, creating a significant challenge: achieving this fidelity at a cost accessible for widespread academic research and training. From a human-factors standpoint, pilots can be viewed as dynamic controllers; the quasi-linear model of McRuer and Krendel [6] remains a benchmark for closed-loop pilot–aircraft analysis. Large-scale simulator work with 110 pilots across 2360 missions demonstrates the value of affordable, repeatable platforms for quantifying such human-control behavior [7]. A critical component for achieving realism in FBW simulation is the control inceptor itself. Active inceptors with programmable force-displacement characteristics have become vital for enhancing situational awareness and providing realistic tactile feedback [8]. A recent survey of force-sensing control technologies for electrically manipulated load systems underscores how inexpensive strain-gauge and capacitive solutions have matured to the point of supporting high-fidelity inceptors [9]. Complementary evidence from mobile-robotics research shows the same cost-resolution trend. Lee et al. [10] report that commodity strain-gauge and capacitive arrays now achieve sub-0.1 % full scale (FS) accuracy while remaining within hobby-grade budgets. This includes the development of various force-feedback sidesticks for flight simulation [11,12] and investigations into active sidestick force control strategies [13]. However, a significant gap exists in the market.

Commercial force-sensing systems that accurately replicate FBW controls are prohibitively expensive, with costs often running into thousands of euros, limiting their widespread adoption in academic and research applications. Conversely, most affordable, non-professional systems rely on position-based sensing, measuring joystick displacement rather than applied force. This fails to replicate the nuanced control feel of a true FBW aircraft and is unsuitable for research on modern control strategies. While various custom joystick designs have been explored in the literature, some focus on co-simulation for flight control systems [14] or using specific load cell systems for interaction force estimation [15], a validated, low-cost, and reconfigurable force-sensing solution specifically for FBW simulation remains largely elusive.

This study addresses this gap by presenting the design, construction, and experimental validation of a low-cost, high-resolution force-sensing sidestick conceived primarily as a diagnostic tool for human factors analysis. While hybrid sidesticks with minimal travel are known to be subjectively preferred [5], our design is intentionally and completely rigid. This choice was made to deliberately remove tactile and proprioceptive cues from small displacements, thereby forcing participants to rely solely on their internal control model and visual feedback. This approach, while creating a challenging control environment, provides a method to assess and differentiate the effects of the underlying control strategies used by the operator. The device uses four strain-gauge load cells to capture pure pilot force inputs with high precision. To our knowledge, no peer-reviewed publication has yet described a strain-gauge sidestick that is both fully open-source and reproducible for under €110 while offering quantified sub-0.1% FS accuracy and human-in-the-loop validation. Previous low-cost efforts-such as the kitchen-scale-based force-feedback joystick proposed by Petković and Đorđević [16], demonstrated the feasibility of using commodity load cells but lacked rigorous calibration, real-time integration with a 6-DoF simulator, or controlled user studies. While early research in active sidestick control explored friction compensation and basic torque feedback using DC motors [17], recent developments have shifted toward high-resolution force-sensing and programmable haptic feedback systems with advanced control algorithms [8]. These modern systems, often featuring optical encoders [16], high-torque actuators, or adaptive force profiles, deliver excellent fidelity but remain prohibitively expensive and technically complex for most academic institutions. By contrast, the sidestick presented here combines €108 in off-the-shelf parts, documented calibration procedures, and a 16-subject evaluation, thereby filling the affordability gap while providing a validated platform for FBW research and training. Accordingly, the sidestick developed in this study is intended as a research platform for human-in-the-loop experiments, enabling systematic investigation of control strategies and pilot workload in flight simulation.

The primary objectives of this work are to: (1) demonstrate the design and implementation of a reconfigurable, affordable force-sensing sidestick suitable for research; (2) evaluate its performance in a dynamic human-in-the-loop flight task with both pilots and non-pilots; and (3) analyze how the device can be used to distinguish different pilot control strategies and their impact on flight performance. The system is integrated into a comprehensive simulation environment featuring a 6-DoF non-linear flight model of a modern fighter airplane in MATLAB/Simulink, co-simulated with X-Plane for real-time visualization [18,19], providing a powerful and accessible platform for future research in human–machine interaction and pilot training applications.

## 2. Materials and Methods

This chapter details the design, construction, and integration of the experimental system used in this study. It is organized into three main sections. First, the design and hardware implementation of the novel force-sensing sidestick are described, including its mechanical structure, sensing elements, and electronics. Second, the calibration process for the sidestick is outlined, covering both linear and non-linear response curves. Finally, the simulation environment and experimental protocol for human-in-the-loop validation are presented.

### 2.1. Sidestick Design and Hardware Implementation

The sidestick developed in this study is specifically tailored to meet high-fidelity simulation demands for academic projects while maintaining low cost. Unlike conventional joysticks that primarily measure displacement, this configuration utilizes force sensing, offering a more intuitive control experience mirroring the force-based logic inherent in modern FBW systems.

**Figure 1:**
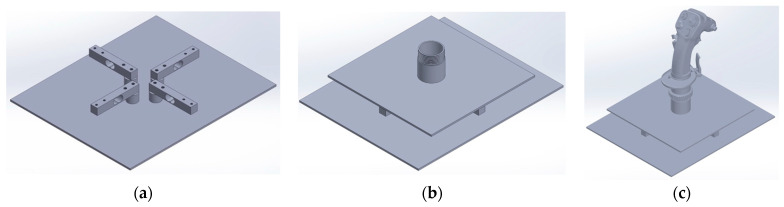
CAD model of the sidestick assembly: (**a**) The lower base plate with four strain-gauge load cells in cross configuration. (**b**) The upper mounting plate transfers grip forces to the cells. (**c**) The fully assembled system with the grip installed.

Structurally, the sidestick incorporates two primary components: a lower base plate and an upper mounting plate. The lower plate anchors the load cells with minimal flex, reducing potential inaccuracies from structural deformation (Figure 1a). The upper plate that is used for transferring forces from the grip onto the load cells is shown in Figure 1b, and the full assembly with the grip installed is shown in Figure 1c.

This dual plate was chosen over alternative approaches due to mechanical robustness and effective isolation of forces along the intended axes, minimizing unintended cross-axis interference. This is achieved because the high rigidity of the upper mounting plate resolves forces and moments applied at the grip primarily into pure shear forces at the four perpendicular load cell locations. This mechanical arrangement effectively prevents torsional loads on any single sensor, which is the primary source of sensor-level crosstalk. Such an arrangement also optimizes mechanical stability, minimizes sensor misalignment, and significantly improves durability while enabling interchangeability of components.

Strain-gauge load cells measure force by detecting small deformations in a metal element. Each cell contains a set of bonded foil strain gauges arranged in a Wheatstone bridge configuration. When a load is applied, the metal element flexes slightly, causing the strain gauges to stretch or compress. These changes alter the electrical resistance of the gauges, unbalancing the bridge and producing a small voltage difference proportional to the applied force. By precisely bonding the strain gauges to a machined, elastic element such as steel or aluminum, the cell delivers highly repeatable, linear responses over its rated capacity [20].

The sidestick employs four strain gauge load cells arranged at 90° intervals: two aligned along the x-axis and two along the y-axis to ensure accurate force measurement.

**Figure 2:**
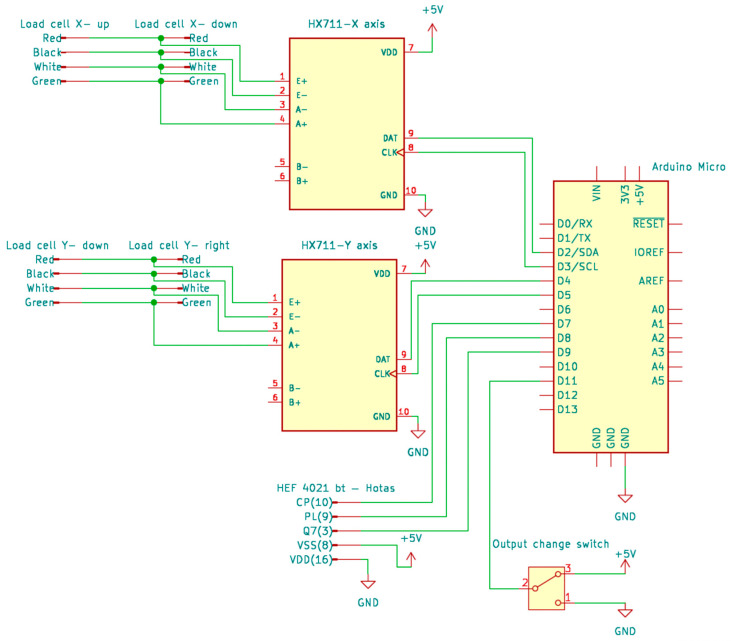
Schematic of the data acquisition electronics, illustrating the parallel connection of load cells for each axis to an HX711 ADC module, which communicates with the Arduino Micro.

To digitize the load-cell signals, we used an HX711 24-bit Analog-to-Digital Converter (ADC) (Tuozhanteng Electronic Technology Co., Ltd., Shenzhen, China) connected to an Arduino Micro (Arduino S.r.l., Turin, Italy). The Arduino was chosen for its built-in USB-HID support, compact size, low cost, and sufficient GPIO. This combination ensures integration and real-time communication with Simulink. The electronics schematic is shown in Figure 2.

The cells of each axis are wired in parallel into the HX711 modules accordingly. Each opposing pair is oriented so that force in one direction produces a positive output and force in the opposite direction produces a negative output when read by the HX711 ADC. A CAD model of this arrangement fixed on the lower plate is shown in Figure 1a.

The load cells, each rated at 10 kg capacity, were chosen to ensure robustness against possible excessive pilot input, thus minimizing the risk of sensor damage due to mechanical overload. The four sensors are single-point aluminum bar-type load cells (model TAL220-10 kg, dimensions ≈ 80 mm × 12 mm × 12 mm). Each cell is factory-calibrated for 98.1 N (10 kgf) with a rated sensitivity of 2.0 mV/V, non-linearity ≤ 0.02% FS, and safe overload of 150%. The full datasheet is provided as Supplementary Material Table S1 to facilitate replication.

The temperature-induced span shift is negligible in this application, since the sidestick operates in a climate-controlled room where ambient temperature varies by no more than ±4 °C. With a nominal sensitivity of 2.0 mV/V and an excitation voltage of 5 V, the full-scale analog output is 10 mV. The HX711’s 24-bit delta-sigma converter, with a fixed PGA gain of 128, digitizes this signal up to 80 Hz.

In practice, the effective number of bits is 20, yielding a quantization step of 0.48 µV, equivalent to a force increment of 0.024 g (0.24 mN) on the 10 kg range. The HX711’s input-referred noise (50 nV RMS) adds an uncertainty of 2.5 µg (0.025 mN) per sample at 5 V excitation.

Combining these error sources in root-sum-square, the theoretical static accuracy is 0.05% FS (0.05 N). This level of precision ensures that the sidestick captures control inputs without perceptible quantization or drift.

The Arduino enumerates as a USB-HID joystick and streams the four force channels to the host PC at 80 Hz (16-bit signed integers), allowing X-Plane to read them as native joystick axes. A custom Simulink block simultaneously ingests the same HID reports, with an estimated end-to-end input latency of ≈12 ± 2 ms. Latency introduced by USB-HID protocols and signal processing can be kept under 20 ms, as demonstrated in similar systems such as the [12].

The Thrustmaster HOTAS Warthog grip (Guillemot Corporation S.A., Carentoir, France) was repurposed as the user interface while using its existing electronics for mapping buttons. Disassembly of the grip revealed that its 19 buttons are routed through three HEF4021BT parallel-in/serial-out shift registers. The serial data (QH), clock (CP), and latch (PL) lines on the PCB were traced, and their connections to the five-pin mini-DIN connector on the bottom of the grip were just like power (VCC) and ground (GND) lines. Arduino code was implemented to assert the PL line, pulse CP to shift each register’s parallel inputs into the serial output, and read the QH line state. The serial bitstream was parsed to determine each button’s status, which was then mapped in software to replicate the original Thrustmaster button indices. This retrofit preserves the grip’s native button functionality and timing characteristics, ensuring that all 19 switches behave identically to the stock configuration.

The vertical distance from the approximate center of the pilot’s grip to the center of the load cells is 170 mm, while the lateral distance from the center of the sidestick to the lower plate anchoring point is 20 mm. The TAL220-10 kg load cells are single-point cells designed to be insensitive to off-center loads within their specified platform size. A maximum pilot input force of 98.1 N (10 kgf) at this height would generate a moment of approximately 16.7 Nm. The robust steel upper mounting plate (Figure 1b) is designed to distribute this moment across the four load cells, preventing torsion on any single cell and keeping the stress well within the manufacturer’s 150% safe overload limit. The successful operation across 16 participants without any sensor degradation confirms the robustness of this design.

### 2.2. Calibration Process

The calibration involved applying known forces in defined increments on each axis of the sidestick [11]. Data from the load cells were recorded using HX711 ADC and processed through the Arduino Micro to establish calibration curves.

**Figure 3:**
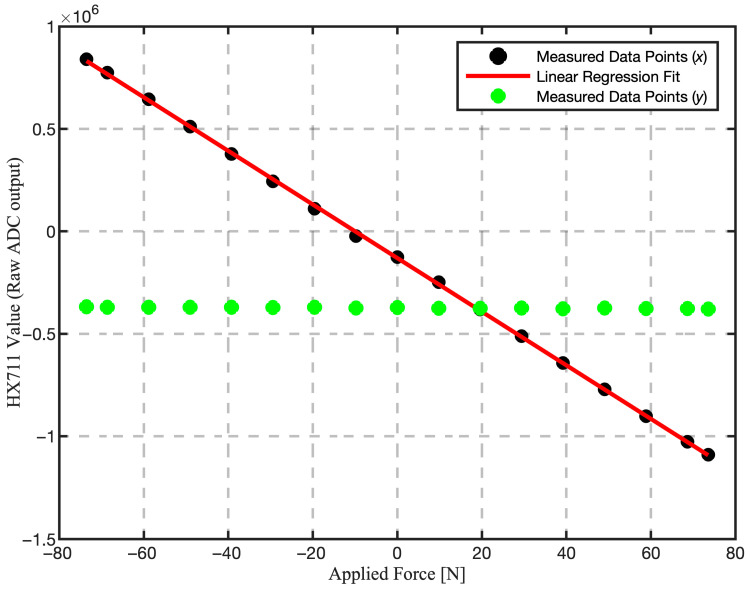
Calibration curve showing sidestick linearity and low crosstalk. Black markers are raw ADC output on the primary *x*-axis versus applied force, with the red linear regression fit. Green markers are the orthogonal *y*-axis output under the same conditions.

To characterize the performance of the integrated sidestick assembly, a static calibration test was performed. Calibrated test masses (from 0 to 7.5 kg) were applied along one axis while simultaneously recording the output from both the primary and orthogonal axes. This method allows for the assessment of system-level linearity and cross-axis sensitivity. The results are presented in Figure 3.

The black data points, representing the output of the assembly on the axis under test (*x*-axis), demonstrate a strong linear response to the applied force. This is evidenced by their tight alignment with the linear regression fit (red line). Crucially, the green data points show the output from the orthogonal (*y*) axis. Their near-constant value confirms that the mechanical design provides excellent isolation between the axes, resulting in minimal crosstalk.

The system-level calibration provided a performance profile of the device, with key characteristics detailed in Table 1. The sensor response demonstrated a robust and predictable linear relationship with the applied force, achieving a coefficient of determination (R^2^) exceeding 0.999. This near-perfect fit confirms that the sidestick reliably translates physical inputs into a consistent electrical signal. The high degree of linearity, complemented by the system’s precision better than 0.05 N and low crosstalk, validates the sidestick’s suitability as a robust tool for capturing detailed pilot inputs in high-fidelity simulations.

Initially, the system was calibrated using a linear model. A 75 N load was applied sequentially in four directions, left and right (*x*-axis) and up and down (*y*-axis), while the Arduino recorded the raw HX711 counts. A baseline measurement, zero force reading, was also taken. Using these data points, a linear approximation was fitted to map sensor outputs into a 0 to 1024 range. On the *x*-axis, 0 corresponds to the maximum left command and 1024 to the maximum right command. On the *y*-axis, 0 corresponds to the maximum up command and 1024 to the maximum down command.

**Figure 4:**
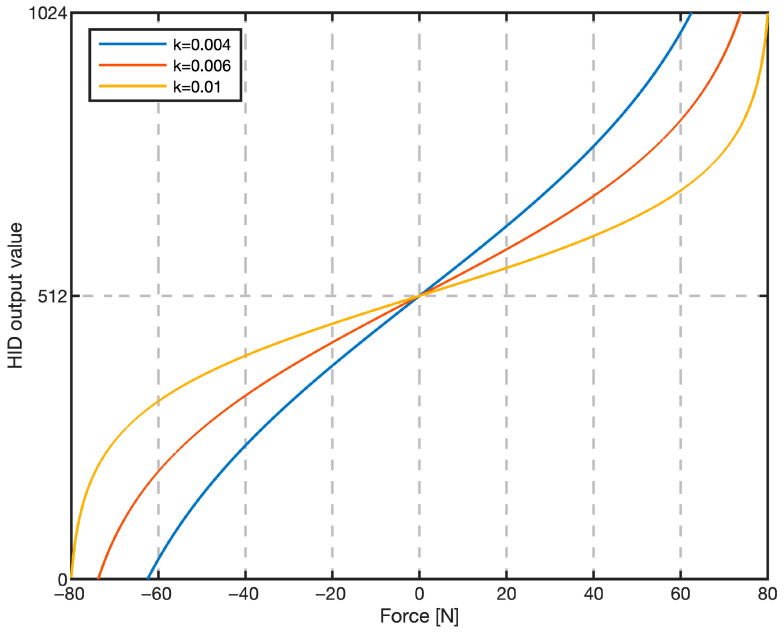
Sigmoid transfer function used for non-linear calibration. Abscissa: input force applied to the grip (−75 N to +75 N). Ordinate: joystick output command (0–1024).

Raw HX711 counts were first normalized to a 0–1 range for firmware processing; however, Figure 4 is plotted in physical force units (N) to illustrate the actual pilot-perceived force.

While the linear model provides a direct force-to-output mapping, a sigmoid-based calibration curve was also implemented to enhance sensitivity near the neutral (zero-force) position while preventing saturation at maximum force.

This non-linear mapping allows for finer, more precise inputs during small corrections, which is critical for tracking tasks. Various steepness values *k* were tested, and *k* = 0.006 was selected for its optimal balance between responsiveness around the neutral position and extrema. The sigmoid mapping increases resolution near zero force, where fine corrections dominate tracking, while the 0–1024 numeric range is retained solely to match a 10-bit HID axis. The sigmoid output function is presented in Figure 4.

A dedicated switch was installed, which allows the user to seamlessly change between the linear and sigmoid output modes during active operation, eliminating the need to disconnect and reconnect the joystick, thus enhancing experimental flexibility.

The 10-bit HID output value (0–1024, as shown on the ordinate of Figure 4) is then linearly mapped to the pitch and roll commands in the Matlab/Simulink R2024b flight model. This connects the pilot’s input with the aircraft’s response. Specifically, the neutral HID value (512, corresponding to the zero-force input after dead-zone) maps to a zero pitch/roll rate command. This implements a “rate-command/attitude-hold” (RCAH) control logic, common in modern FBW aircraft, where the aircraft maintains its current attitude when the stick is neutral. Full-scale deflection of the HID output (0 or 1024) corresponds to the maximum permissible pitch and roll rates defined in the aircraft’s flight dynamics model. This two-stage mapping, first from physical force to a digital HID value via calibration curves, and then from that value to a state-rate command, is fundamental to replicating the control logic of the simulated FBW system.

To prevent the stick from reacting to the static weight of the operator’s hand, the firmware applies a symmetric force dead-zone of ±4 N. Forces whose magnitude falls below this threshold are set to zero, thereby suppressing micro-motions and physiological tremors while the hand merely rests on the grip. The 4 N limit, roughly 5% of full scale, was determined empirically as the smallest value that eliminated inadvertent inputs across all participants without masking intentional control forces. Future firmware will allow the dead-zone to be user-programmable to accommodate individual preference and grip mass.

**Figure 5:**
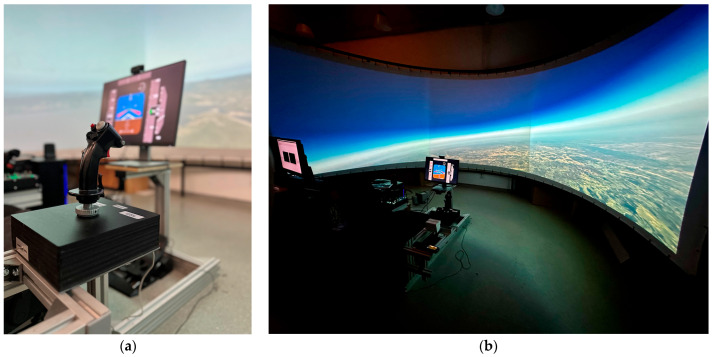
Experimental setup and sidestick assembly. (**a**) Assembled force-sensing sidestick with integrated load-cell mechanism and modified HOTAS Warthog grip in the user test configuration. (**b**) Human-in-the-loop flight simulator environment with panoramic projection screen and sidestick test station visible in the foreground.

The completed and fully assembled sidestick, shown in practical use in real testing conditions, is shown in Figure 5.

This sidestick is cost-effective, modular, and provides a high-resolution control interface. Table 2 presents the cost breakdown, listing the price of each component. The Thrustmaster Warthog grip is not included, as it was already available and did not need to be purchased. The total cost of this force-sensing sidestick is EUR 108, whereas comparable off-the-shelf solutions range from EUR 1500 to EUR 4000 (excluding the grip), i.e., [21]. This significant price reduction makes force-sensing joystick research accessible at a fraction of the usual cost. These characteristics align closely with the stated objectives of the broader research initiative, namely, to develop a robust, realistic, and adaptable training platform capable of accurately simulating modern fly-by-wire sidesticks found in aircraft.

### 2.3. Experimental Protocol

#### 2.3.1. Research Flight-Simulator Facility

The experiment was conducted on the research flight-simulator facility operated by the Department of Aeronautical Engineering, Faculty of Mechanical Engineering and Naval Architecture (Zagreb). This fixed-base simulator uses two linked PCs: one executes the 6-DoF aircraft model in MATLAB/Simulink, while the other renders out-the-window visuals with X-Plane 11 and instruments via Air Manager 5. Visuals are projected by three XGA projectors onto a 2.5 m-radius cylindrical screen spanning 180° horizontally and 50° vertically (Figure 5). A metal cockpit frame carries a seat, the force-sensing sidestick, throttle quadrant, rudder pedals, and a 15 in instrument monitor. The facility is routinely employed for handling quality studies of UAVs, general aviation aircraft, and jet fighters, and therefore provides an appropriate, high-fidelity environment for the present human-in-the-loop tests.

#### 2.3.2. Participants

Sixteen volunteers participated: 6 licensed civil pilots and 10 non-pilots recruited from the university community. Demographic and flight experience data are summarized in Table 3. The median age of the group was 25.5 years, with participants ranging from 22 to 54 years old. Pilots’ flight experience ranged from 15 h to 800 h (median = 142 h), whereas non-pilots reported no certified flight time. Simulator exposure varied widely (from 1 h to 500 h, median = 23 h). Simulator hours includes all the time everyone spent in any flight simulator before this experiment. All participants were right-hand dominant and reported being free of musculoskeletal disorders that could impair control operation. Furthermore, all participants confirmed having normal or corrected-to-normal vision, ensuring they could effectively interpret the visual displays throughout the experiment.

#### 2.3.3. Task and Procedure

To validate the diagnostic sensitivity of the developed FBW sidestick, an initial proof-of-concept study was conducted. Flight test was initiated from level flight at 90 m/s speed and 3000 m altitude while participants performed a pitch angle tracking task. This type of continuous tracking task was specifically chosen over discrete maneuvers, such as landing, as it provides a highly repeatable, standardized stimulus and generates a continuous data stream ideal for analyzing control strategies and human operator dynamics. The primary goal of this validation was not to draw definitive conclusions about pilot skill, but to demonstrate the device’s capability to differentiate between distinct, measurable patterns of control input under a standardized, demanding task. The target signal for tracking was constructed as a sum of sinusoidal components, a standard approach in pilot modeling studies. This “sum-of-sines” tracking paradigm is a well-established methodology for creating a complex, yet repeatable, pseudo-random command signal, making it ideal for analyzing a pilot’s control behavior and handling qualities in a closed-loop system [6,19,22,23,24]. The use of such composite signals for human-in-the-loop flight simulation has been extensively validated in prior work, including studies foundational to this research [19,24]. The signal is defined as the sum of sinusoidal forms according to Equation (1).

(1) $$\Theta_{i}(t)=\sum_{j=1}^{n}\;A_{j}\;\sin\left(\omega_{j}\cdot t+\phi_{j}\right)+\Theta_{0}$$

where

- $\Theta_{i}(t)$ is the time-varying reference pitch angle (in degrees);
- $A_{j},\;\omega_{j}$, and $\phi_{j}$ represent the amplitude, frequency, and phase shift in each component;
- $\Theta_{0}$ is an offset added to emulate the nominal trimmed pitch attitude of the simulated aircraft.

This type of composite signal was adapted from prior work in human-in-the-loop control studies [19,22] and designed to produce an overall signal. The complete commanded pitch angle presented to the user was thus defined as

(2) $$\Theta_{c}\left(t\right)=\Theta_{0}+\Theta_{i}\left(t\right)$$

An objective measure of tracking performance was defined as the error signal $e\left(t\right)=\Theta_{i}\left(t\right)-\Theta \left(t\right)$, i.e., the difference between the commanded and actual pitch angle $\Theta \left(t\right)$. The tracking error variance was used as the primary performance metric, as it reflects the pilot’s precision in following the reference trajectory. Because the command signal appears pseudorandom and unpredictable (despite being deterministic), it increases task complexity and makes the tracking variance a more meaningful metric than raw final accuracy or offset. This approach is standard in man-in-the-loop control testing, where consistent reference complexity is required across trials.

**Figure 6:**
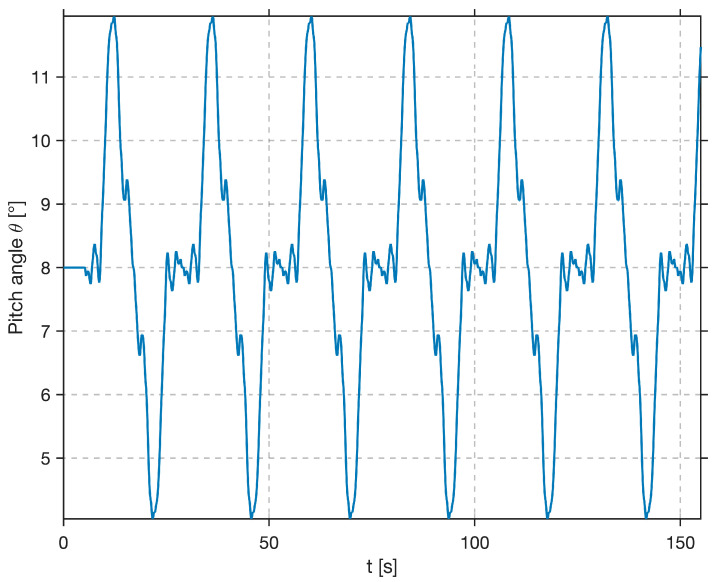
Time history of composite reference signal $\Theta_{c}\left(t\right)$ for the pitch-tracking task (offset by 8° for trim).

To emulate the initial trim and allow for pilot reaction time, the excitation signal remained constant at 8° for the first 5 s before transitioning into the dynamic sum-of-sines profile. The complete reference signal, shown in Figure 6, ranges from 4° to 12°, with a mean $\Theta_{0}=$ 8° for chosen initial conditions.

**Figure 7:**
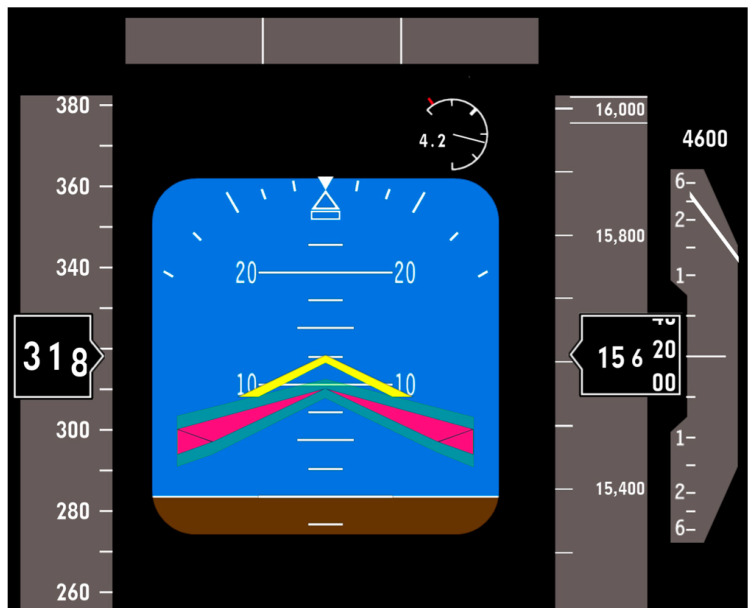
The Electronic Attitude Director Indicator (EADI) display used during tracking: the yellow symbol shows current pitch attitude, the magenta symbol the target. Participants aimed to align the two throughout the task.

For the implementation of the signal in the 6-DoF simulator of fighter aircraft, the Air Manager software package was used, and an existing indicator was adapted. The Electronic Attitude Direction Indicator (EADI) employed during the execution of the first task is shown in Figure 7.

This protocol emulates human-in-the-loop pilot-evaluation scenarios commonly employed in the literature [19,22,23,24]. The magenta triangle vertices represent the commanded pitch profile $\Theta_{i}\left(t\right)$ defined in Equation (1), while the vertex of the yellow arrow shows the aircraft’s actual pitch Θ(t). A translucent green band surrounds the magenta trace to visualize the permissible tracking error *e*(*t*). Participants were instructed that successful task performance is achieved when the yellow arrow remains inside the green tolerance envelope for the 155 s trial.

Before beginning the experiment, each candidate was given ten minutes of “free flight” so that they could familiarize themselves with the simulator’s flight controls and the aircraft’s dynamic response. Control inputs from the sidestick were interpreted as pitch rate commands, consistent with the RCAH logic, requiring participants to integrate this rate command over time to achieve and hold the target pitch angle. Before the task, the candidate received verbal instructions detailing the specific objective of the task as well as its duration. After the 10 min familiarization, each participant completed one 155 s tracking run using the identical reference signal in Figure 6; no additional runs or signal variations were administered. Each run started from steady, level flight at an altitude of 3000 m and a true airspeed of *V* = 90 m/s. The aircraft was intentionally left untrimmed so that participants could adjust longitudinal trim at their discretion. The throttle (gas setting) was likewise pilot-controlled throughout the trial. During all experimental runs, the sidestick operated in sigmoid-response mode because preliminary testing showed this mapping provided finer control near neutral than the linear curve.

The results were evaluated using two complementary approaches: objective metrics and subjective assessments. The objective workload analysis evaluated candidate performance using two methods. The first method compared the variance of the tracking error between the prescribed pitch-angle reference signal and the actual achieved pitch-angle throughout the task. The tracking error variance was computed according to Equation (3).

(3) $${Var}_{e}=\frac{1}{N-1}\sum_{i=1}^{N}{\left|e-\mu \right|}^{2}\;$$

where

- *N* is the total number of samples,
- *μ* is the mean value of the difference.

The second objective workload metric was based on counting the number of local maxima and minima (extrema) in the stick force input signal [25]. This metric provides quantitative information regarding the physical and mental effort exerted by the candidate during the task. In addition, the time derivative of the stick force was analyzed. It was obtained by differentiating the stick force signal with respect concerning time, to quantify the abruptness of force changes. Plotted alongside these metrics were additional flight parameters, including airspeed, altitude change, pitch rate variation, and angle-of-attack variation. Observing how these parameters evolve concurrently offers insight into a candidate’s ability to simultaneously control multiple flight variables.

Subjective evaluation was conducted using two well-established methods: the Cooper-Harper handling qualities rating scale (CHR) and NASA Task Load Index (NASA-TLX). The Cooper-Harper scale is a standardized, decision-tree-based tool used extensively in flight testing. It guides the rater to a final 1-to-10 score that quantifies the handling qualities of an aircraft in the context of the effort required by the pilot to achieve a desired level of performance [26]. The NASA-TLX, conversely, is a multi-dimensional instrument designed to provide a more diagnostic assessment of perceived workload. It separately measures six factors: Mental Demand, Physical Demand, Temporal Demand, Performance, Effort, and Frustration, to create a comprehensive profile of the sources of workload, rather than a single global score [27].

After the flight task, participants rated handling qualities using the Cooper-Harper scale, and workload using NASA-TLX (rating mental, physical, temporal demand, etc.) on 0–20 scales, where lower values indicate lower perceived workload and higher values indicate higher perceived workload.

After the experimental session, candidates were asked to provide open-ended feedback on their impressions of the new fly-by-wire control stick and to suggest improvements for enhancing handling quality.

We also planned statistical comparisons using a two-sample *t*-test for pilot vs. non-pilot performance (with checks for normality and equal variances), and we computed Pearson correlation coefficients to examine relationships between objective and subjective metrics.

## 3. Results

### 3.1. Objective Analysis

**Figure 8:**
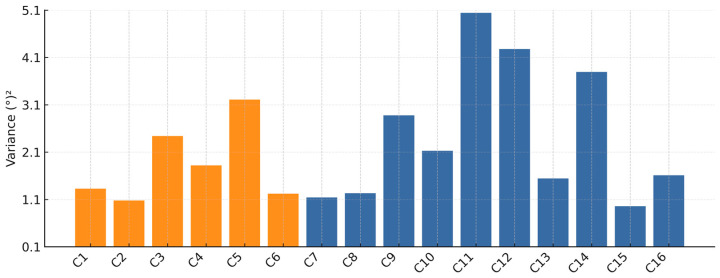
Tracking error variance for all 16 participants. Pilots (orange bars) exhibit lower and more consistent variance compared to non-pilots (blue bars).

Figure 8 displays tracking-error variances in (°)^2^ for all sixteen participants, labeled C1 through C16 on the horizontal axis. The vertical axis quantifies variance in (°)^2^ computed using Equation (2). The orange bars represent the pilot group, whose flight experience ranged widely from student-pilot to commercial-pilot levels (15 h to 800 h), a factor that contributes to the observed variance. Orange bars (pilots) are relatively uniform and low, demonstrating consistently tighter tracking. Blue bars (non-pilots) show greater dispersal.

While some non-pilots (e.g., C7, C8, C13, C15, C16) achieve low variances like pilots, others (especially participants C11, C12, and C14) exhibit large variances, indicating less accurate pitch-angle control. In summary, the chart shows that candidates with piloting experience generally maintain error variances between 1.08 (°)^2^ and 3.21 (°)^2^, while non-pilots span from 0.95 (°)^2^ to 5.04 (°)^2^, with the highest and lowest single variances both occurring in the non-pilot group. Participant C15 has the lowest variance of 0.96 (°)^2^, indicating the most precise tracking among all participants. Participant C11 has the highest variance of 5.04 (°)^2^, indicating the poorest tracking performance.

**Figure 9:**
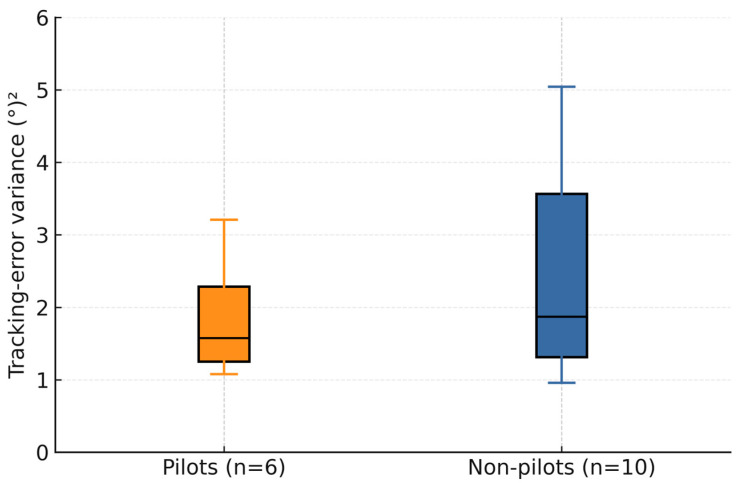
Box plot of tracking error variances, comparing pilots and non-pilots.

In Figure 9, the variance box plot is presented comparing pilots and non-pilots. Although the median track error variance is nearly the same for pilots 1.57 (°)^2^, and non-pilots 1.87 (°)^2^, the pilot group exhibits a much more compact distribution of variances, as evidenced by the smaller interquartile range (IQR) of 1.24 (°)^2^ and narrower whiskers. This means that pilots’ performance was more consistent, and most pilots fell within a relatively tight band of tracking-error variances. In contrast, the non-pilot group shows a substantially larger spread of IQR around 2.60 (°)^2^.

Some non-pilots achieved low variances comparable to the best pilots, but several non-pilots also exhibited very high variances. The taller blue box and longer whiskers illustrate that non-pilot performance was far more variable. Some non-pilots tracked as well as pilots, while others tracked poorly. Participants with piloting experience demonstrated more predictable and tightly clustered tracking performance, whereas non-pilots ranged from excellent to very poor, resulting in much wider dispersion of their error variance values.

**Figure 10:**
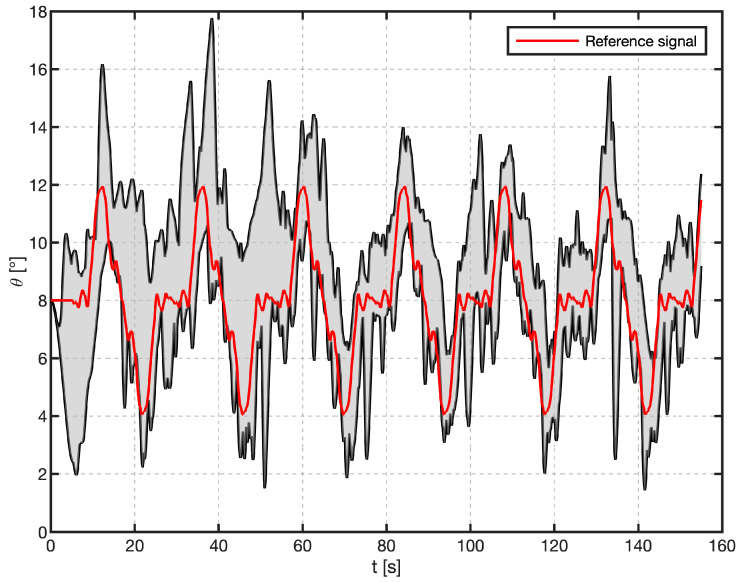
Envelope of pitch-angle responses for all 16 participants. The red line is the reference signal. The black lines represent the maximum and minimum of actual pitch angles achieved by all participants at each point in time, and the shaded gray area between them illustrates the full range of responses.

In addition to quantifying error variance, the span of achieved pitch angles provides a complementary metric for evaluating task performance. Figure 10 depicts the envelope of pitch-angle responses. For each time step, the minimum and maximum recorded pitch angles $\Theta \left(t\right)$ were identified, and their difference, which represents the instantaneous response range, is shaded in gray. The red curve corresponds to the reference signal that the subjects were instructed to track $\Theta_{i}\left(t\right)$.

The pitch angle spans from approximately 2° to 18°, while the reference signal is constrained between 4° and 12°. At the envelope’s extremities, individual pitch angle deviations reach magnitudes of roughly ±6° relative to the reference signal. During the first few seconds of the trial, the angular measurements span a wider range as participants initially adjust and attempt to ‘catch’ the plane. Once they settle into the tracking task, the variability decreases, and the signal converges toward the target trajectory.

**Figure 11:**
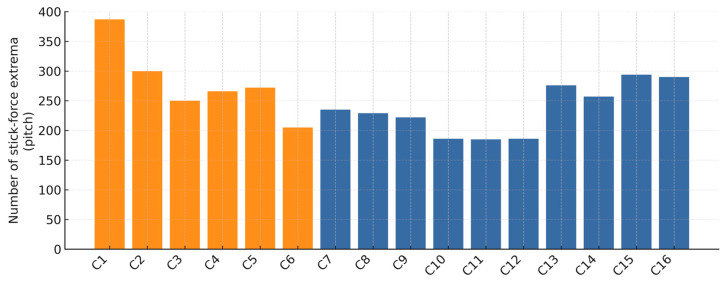
Stick force extrema count (maxima and minima) per participant. Higher values indicate more active control. Pilots (orange) generally show more frequent control reversals than non-pilots (blue).

In Figure 11, the count of stick force extrema is shown, which serves as an indicator of the subject’s physical effort during aircraft control. A higher number of extrema implies that the applied force is reversed more frequently, reflecting greater physical exertion.

To quantify the relationship between control activity and tracking performance, a Pearson correlation analysis was conducted between the stick force extrema count and the tracking-error variance for all 16 participants. The analysis revealed a strong, statistically significant negative correlation (*r* = −0.68, *p* = 0.004). This indicates that participants who made more frequent control inputs (higher extrema count) generally achieved lower tracking-error variance, suggesting a link between a more active control strategy and higher precision. Pilots generally applied more micro adjustments, but a few non-pilots (notably C15) matched or exceeded that level of stick-force activity, which aligns with their superior tracking performance. In contrast, participants with very low extrema counts struggled to follow the reference signal and ended up with higher error variances. Pearson correlation analysis showed no statistically significant linear relationship between prior simulator hours and tracking-error variance among non-pilots (*r* = −0.44, *p* = 0.21).

Next, the results for the best performer (C15) and the worst performer (C11) are presented.

**Figure 12:**
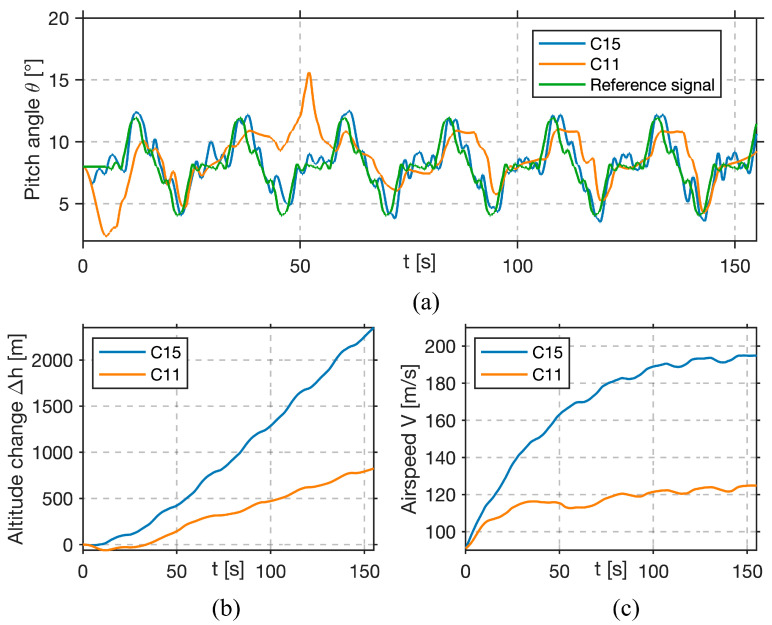
Flight data for the best (C15) and worst (C11) performers. (Top) (**a**) C15’s pitch angle (blue) closely tracks the reference (green); C11 (orange) shows lag and large deviations. (**b**) C15 steadily gains 2200 m altitude; C11 only 800 m with an erratic profile. (**c**) C15 maintains a smooth airspeed increase; C11 shows poor airspeed control.

In Figure 12a, the green curve represents the reference pitch-angle signal, and the blue curve shows the best-performing participant’s (C15) actual pitch angle. It can be observed that the actual pitch angle remains tightly bound to reference signals, with only minor deviations, most of which are at the start and end of the experiment. For the worst-performing participant C11, depicted with the orange curve, it can be observed that the actual pitch angle exhibits large deviations. For large portions of the task, C11 fails to track the reference signal, often lagging by multiple seconds or overshooting by several degrees. In Figure 12b altitude change is shown. The best-performing candidate, depicted by the blue curve, has a relatively steady climb of approximately 2200 m, which indicates that pitch commands were coordinated with throttle inputs, thus enabling steady climb. For the worst-performing candidate (orange curve), a climb of 800 m can be seen, which is significantly less than the candidate’s C15 altitude change.

On the bottom right graph in Figure 12c, airspeed is shown. The best-performing candidate (blue curve) achieved a smooth and consistent speed increase of nearly 100 m/s. The worst-performing candidate (red curve) exhibited a slower and uneven speed profile, which, combined with the altitude change, suggests poor coordination of throttle inputs during the experiment.

**Figure 13:**
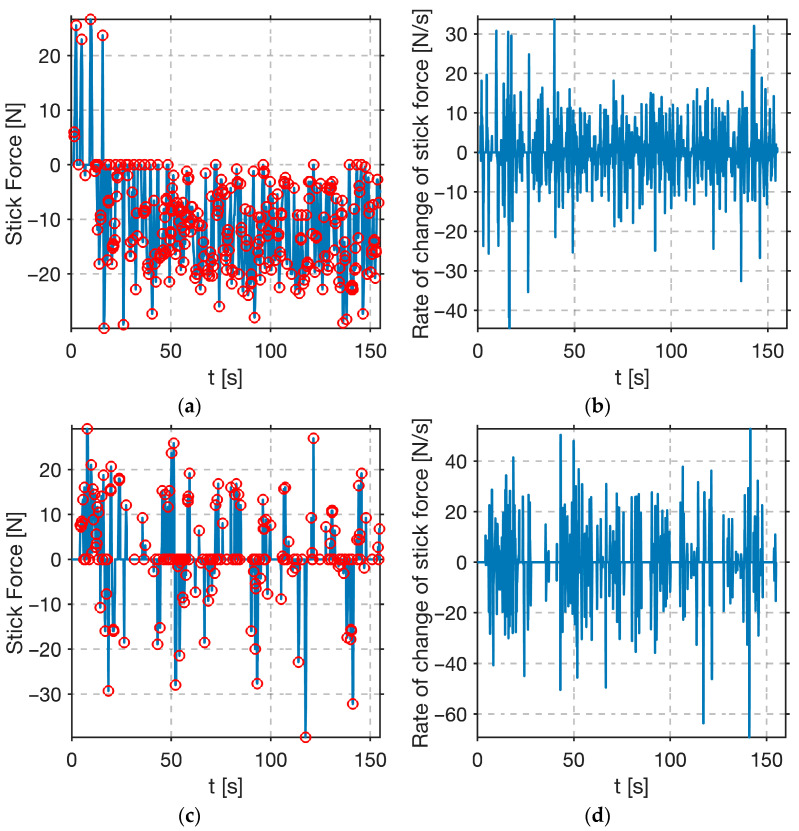
Control input data for the best (C15) and the worst (C11) performers. (**a**,**b**) C15 shows sustained pulling force with smooth, frequent micro-adjustments, typical of feedforward control. (**c**,**d**) C11 exhibits wide oscillations and abrupt spikes, consistent with reactive, error-based input.

In Figure 13, stick force and rate of change in stick force for both candidate’s flights are shown. Stick force for the best-performing participant (Figure 13a) exhibits a sustained negative bias, a slight, continuous pull during the ascent phases, with only minor positive inputs applied during descent. Most stick force extrema counts occur in the negative force domain, and this pattern reflects a predominantly feedforward approach: the candidate maintains back pressure to preload the control surfaces and then makes fine force reversals around zero only when necessary.

In Figure 13c, the graph of stick force for the worst-performing participant is shown. The stick force oscillates around zero, with many zero crossings, which mirrors a conventional, displacement-based stick usage. There are significantly fewer extrema than those observed in C15. Meaning that zero force is maintained until the error threshold is exceeded, then the candidate applies a large corrective action, which results in overshooting the reference signal.

In Figure 13b, the graph rate for the change in stick force for the best-performing participant is shown. Oscillations are uniform in amplitude, typically bound within ±15 N/s for most of the experiment, with occasional spikes up to ±40 N/s near zero-crossings of the reference signal. The consistent amplitude of these derivative oscillations indicates smooth adjustments rather than abrupt movements.

In Figure 13d, the rate of change in stick force for the worst-performing participant is shown. During the experiment, there are frequent spikes of ±35 N/s, which are much higher than those in the participant’s C15 results. Such high derivative magnitudes indicate that, when participant C11 decided to correct an error, he did so with a sudden, forceful stick reversal rather than a gradual adjustment.

### 3.2. Subjective Analysis

**Figure 14:**
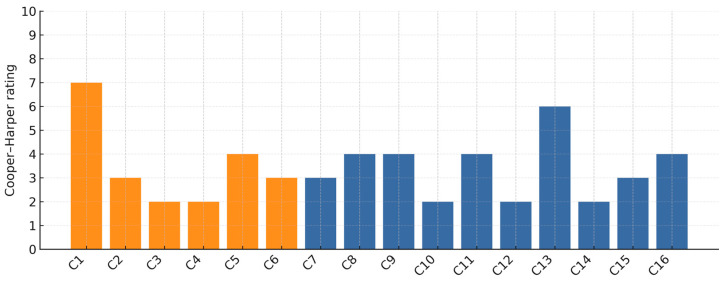
Subjective Cooper–Harper handling qualities ratings for each participant. Lower scores (1–3) indicate better perceived handling qualities, while higher scores (7–10) indicate poor handling.

Figure 14 displays each participant’s Cooper–Harper rating for the specified aircraft and tracking task. Ratings 1–3 denote Level I (satisfactory) handling qualities, 4–6 correspond to Level II (adequate with noticeable pilot compensation), 7–9 fall into Level III (marginal—large compensations required), while a rating of 10 indicates the aircraft is effectively uncontrollable for the task. Two colors distinguish candidates with piloting experience (orange bars) from non-pilots (blue bars). The results show a general correlation with the objective performance metrics (Figure 8 and Figure 11).

For 10 out of 16 participants, there is alignment between subjective Cooper–Harper ratings and objective tracking errors. Participants C2, C6, C7, and C15 each achieved error variances below 1.3 (°)^2^ and rated Cooper–Harper as 2 or 3. Their control strategies (continuous micro adjustments) minimized variance and translated into minimal perceived difficulty. Participants C5, C9, and C11 demonstrated larger variances, >2.9 (°)^2^, and rated Cooper–Harper as 4, indicating that they recognized the need for compensatory inputs. Their reactive control strategies generated measurable error and a moderate sense of workload.

Two clear outliers illustrate mismatches between perception and performance. Participant C1 recorded a low error variance of 1.33 (°)^2^ yet assigned a relatively poor CHR rating of 7, indicating the task felt difficult despite accurate tracking, whereas C12 showed a high variance of 4.28 (°)^2^ but self-rated CHR = 2, suggesting an over-optimistic appraisal.

Across groups, pilots tended to cluster at the favorable end of the scale (median CHR = 3). The single worst rating (7) was also from a pilot, though the sample size precludes firm group-level conclusions.

**Figure 15:**
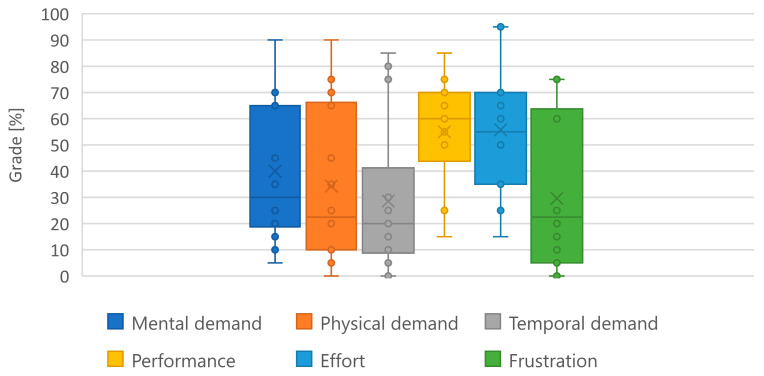
Mean NASA-TLX workload scores across all participants, normalized to a percentage scale. The six subscales (Mental, Physical, Temporal Demand, Performance, Effort, and Frustration) provide a multi-faceted assessment of subjective workload experienced during the task. Scores are raw (unweighted) NASA-TLX ratings, linearly rescaled to a 0–100% axis for visual comparison (20 = 100%, 0 = 0%).

In Figure 15, participants’ NASA-TLX scores for the task are shown. The plotted scores have been normalized to percentages, with a raw score of 20 corresponding to 100% and a raw score of 0 corresponding to 0%.

Physical demand exhibited the widest spread (0–90%, median ≈ 35%), indicating that some participants found the required stick forces trivial while others found them strenuous. Temporal demand remained low (median ≈ 20%), whereas Effort showed a notably high median (≈55%) with a few extreme reports approaching 95%, suggesting that certain participants had to work very hard to maintain control. Frustration scores were generally modest (median ≈ 30%), although a handful of outliers reported high frustration.

Participants employed a range of control strategies, evident in their variance scores and stick input profiles. Table 4 compares the best (C15) and worst (C11) performers, revealing large differences in tracking precision, workload, and stick behavior.

As shown in Table 4, the best performer used a proactive, feedforward control strategy, achieved substantially lower error variance, 0.96 (°)^2^ vs. 5.04 (°)^2^, exerted more fine-grained control (higher extrema count), and reported dramatically lower workload (50% vs. 95% Effort). These contrasts underline the sidestick’s sensitivity to distinct control paradigms.

A Pearson correlation of *r* = 0.87 (*p* < 0.001) between Cooper–Harper ratings and error variance substantiates the Cooper–Harper scale’s sensitivity.

Interestingly, effort scores from the NASA-TLX did not significantly correlate with tracking-error variance (*r* = 0.17, *p* = 0.52), suggesting that participants’ perceived exertion was not always aligned with their objective performance. This discrepancy highlights the multifactorial nature of perceived workload, which may reflect cognitive strain or control strategy rather than tracking precision alone. This also aligns with the observed outliers, such as C1 and C12, whose subjective effort did not match their measured error, indicating that effective performance can sometimes be achieved through strenuous control, and vice versa.

### 3.3. Group Statistics

Group-level differences in error variance were evaluated as follows: Shapiro–Wilk tests confirmed normality (pilots: *W* = 0.89, *p* = 0.32; non-pilots: *W* = 0.88, *p* = 0.14), and Levene’s test indicated equal variances (*F* = 1.36, *p* = 0.26). A two-sample *t*-test found no significant group difference (*t* = −0.93, *p* = 0.37), although the moderate effect size (Cohen’s *d* ≈ −0.48, 95% CI [−1.74, 0.51]) and low achieved statistical power (~0.44 at α = 0.05) suggest that detecting such an effect would require approximately 34 participants (≈17 per group) for 80% power (Lehr’s approximation).

## 4. Discussion

### 4.1. Control Strategies

Our results demonstrate that control strategy, rather than prior flight experience, was the primary determinant of performance on the low-cost strain-gauge sidestick. We identified two distinct control paradigms: a proactive, high-frequency, low-amplitude feedforward strategy that yielded low tracking error, and a reactive, low-frequency, high-amplitude feedback strategy that resulted in poor performance and significant phase lag.

Notably, pilots did not uniformly outperform non-pilots; instead, participants adopting an anticipatory feedforward strategy, regardless of flight experience, achieved lower error and reported lighter workload.

The divergence between the best (C15) and worst (C11) performers, detailed in Table 4 and Figure 14, starkly exemplifies this. The strong negative correlation between extrema counts and error variance (*r* = −0.68), reported in the results, is embodied by these two participants. Participant C15, using a proactive approach, sustained a negative force bias and made 295 frequent, low-amplitude micro-adjustments with smooth derivative profiles (±15 N/s). This strategy produced a low tracking variance of 0.96 (°)^2^, minimized phase lag, and enabled effective energy management, resulting in a steady 2200 m climb. In contrast, C11’s reactive control resulted in an error variance over five times higher, 5.04 (°)^2^. With only 185 large, abrupt adjustments (up to 35 N/s), C11 struggled with erratic airspeed control and achieved only ~40% of C15’s altitude gain. Such a clear distinction in control techniques, made visible through measurable micro-movements, demonstrates that the sidestick’s utility extends beyond mere control; it functions as a diagnostic tool.

This contrast demonstrates that the sidestick’s utility extends beyond mere control; it functions as a diagnostic tool. Its high resolution and low noise floor are what make it possible to reliably measure the subtle micro-movements that differentiate control techniques, thus revealing suboptimal strategies. These findings align with established models of human operators as dynamic systems, where the choice of control strategy is known to significantly impact system stability and performance [28].

### 4.2. Correlation Subjective/Objective

Tracking-error variance was strongly associated with Cooper-Harper ratings (*r* = 0.87, *p* < 0.001), confirming the scale’s validity for assessing handling difficulty. In contrast, NASA-TLX Effort did not show a significant correlation with variance (*r* = 0.17, *p* = 0.52), suggesting perceived exertion reflects factors beyond pure tracking performance. This finding may be partly explained by the inherent nature of the completely rigid control stick. While true to some FBW systems, this design lacks the minimal physical travel that provides crucial tactile cues. Consequently, the reported effort might not only reflect the workload of the tracking task itself, but also the cognitive and physical strain of adapting to this “unnatural” control interface. Outliers highlight this complexity: some candidates with low variance reported high workload (effortful precision), while others with high variance understated difficulty (poor error awareness). This trend mirrors Jalovecký’s [7] findings of wide inter-individual variability even in standardized tasks, reinforcing the need for open-access, strategy-sensitive tools like the presented sidestick. These discrepancies also highlight the necessity of combining objective performance data with subjective feedback to obtain a holistic picture of human–machine interaction.

### 4.3. Group Comparison

The lack of a statistically significant difference in mean tracking error between the heterogeneous pilot and non-pilot groups (*p* = 0.37) is, in itself, an important finding. It strongly suggests that in a highly specific, visually dominated tracking task with an unfamiliar rigid inceptor, generic piloting experience is less important than the individual’s ability to adapt and apply a viable closed-loop control strategy. While pilots as a group were more consistent (lower IQR), the performance overlap indicates that this sidestick effectively measures task-specific skill rather than general aviation expertise.

The moderate effect size (Cohen’s *d* = −0.4819) suggests that with a larger sample, a significant difference might emerge. Given the moderate effect size (*d* ≈ −0.48) and low achieved power (≈0.44), future studies should recruit at least 34 participants (17 pilots, 17 non-pilots) to reliably detect inter-group differences in tracking performance. The true value of the sidestick, therefore, lies not in differentiating coarse groups but in its sensitivity to capture performance differences at the individual strategy level, which may be more granular than a simple pilot/non-pilot dichotomy.

### 4.4. Design Implications and Next Steps

While other custom joysticks have been developed, they often involve more complex mechanisms or serve different purposes, such as fuzzy logic control or combined force/position sensing. A recent review on force sensing control and key technologies in manipulated load systems further highlights the ongoing challenges in achieving realistic and affordable force feedback [9]. Previous low-budget attempts, e.g., Petković and Đorđević’s kitchen-scale joystick [16], proved that commodity load cells can sense pilot forces, but lacked rigorous calibration or human-in-the-loop validation. At the other extreme, optical inceptor sensing [28] and high-torque active sticks [17] achieve superb fidelity yet at far higher complexity and cost.

Our system’s sub-0.1% FS static accuracy, achieved at a very low cost, positions it uniquely in the literature. By leveraging off-the-shelf components and open-source electronics, this sidestick fills the gap between basic gaming controllers and professional aerospace-grade inceptors. Its high-fidelity, low-latency force-sensing enables advanced research in fly-by-wire control laws and human factors at a fraction of the cost of commercial force-feedback systems. This accessibility promotes broader academic adoption, supports pilot training programs, and accelerates the development of next-generation control interfaces.

By open-sourcing the design principles, we provide a platform that can be replicated and extended by other researchers, democratizing access to high-fidelity FBW control research.

### 4.5. Benchmark Comparison

To provide a performance context for our novel device, it is useful to reference results from a separate study that used an identical tracking protocol with a commercial, displacement-based controller (Thrustmaster HOTAS Warthog) [29]. It is critical to note that this comparison is purely indicative for establishing a rough performance baseline, as the participant cohorts differed substantially. The benchmark study involved a homogeneous group of low-hour student pilots (median = 10 h), whereas the pilot group in the present study had significantly more experience (median = 142 h).

In that benchmark study, the median tracking-error variance was 1.8 (°)^2^ (IQR = 2). For context, the median variance for the experienced pilot group in our study was 1.57 (°)^2^ (IQR = 1.24). This suggests that the overall tracking accuracy achieved with our rigid, force-sensing system is within a comparable order of magnitude to that of a high-fidelity displacement-based controller, even with the added challenge of a non-intuitive interface. This provides confidence that the device functions as a valid and effective control inceptor for this type of task.

### 4.6. Limitations

The study involved a small, heterogeneous sample (*N* = 16), a single-axis task, and one 155 s tracking run per participant. While a single-trial design simplified scheduling, it precluded estimation of intra-subject reliability and the use of repeated-measures statistics. In upcoming work, we will adopt a familiarization–baseline–test protocol with ≥3 runs per participant, providing data for within-subject ANOVA and test–retest analysis.

Future studies will also include multi-axis scenarios, larger cohorts, and programmable force-deflection gradients to assess handling quality improvements.

Equally important, the pilot cohort itself was not homogeneous: total logged flight time ranged from 15 h to 800 h, spanning student-pilot to commercial-pilot proficiency. Such variability almost certainly dilutes any group-level expertise effect and may explain why the mean tracking error of pilots did not differ significantly from that of non-pilots.

To address this, forthcoming experiments will employ a stratified sampling frame with pre-defined experience bands (e.g., <50 h, 50–300 h, >300 h) or match each pilot with a non-pilot of similar simulator exposure. This design will sharpen statistical power for detecting genuine expertise-related differences while controlling for simulator familiarity.

The most consistent piece of subjective feedback was the “unnatural” feel of a completely rigid stick. A similar issue arose in early F-16 development, where a fully rigid sidestick proved “too sensitive”; engineers ultimately introduced ~6 mm of artificial travel to improve feel and reduce cross-axis coupling [4]. This suggests that while pure force sensing is true to some FBW systems, a minimal amount of physical displacement or haptic feedback may nonetheless be crucial for user acceptance and performance. This aligns with Rummel’s equivalency analysis [5], where a hybrid stick outperformed a rigid force-only stick in subjective handling qualities. Research consistently shows that inceptor characteristics fundamentally shape pilot interaction and performance [3]. Furthermore, this observation provides a compelling explanation for the decoupling of perceived effort (as measured by NASA-TLX) from tracking performance, as observed in our results. The struggle to adapt to a control interface devoid of familiar tactile motion cues likely contributed to the reported physical and mental demand, irrespective of the final tracking accuracy. Therefore, despite this design choice being a deliberate trade-off between cost and simplicity, on the one hand, and haptic realism, on the other, future research will focus on integrating small-displacement haptic actuators to provide programmable force-deflection gradients. We also plan to implement user-selectable display conventions and refined control response curves as suggested by participants and conduct a comparative study to quantify the handling quality improvements.

## 5. Conclusions

This study has successfully designed, built, and validated a low-cost, reconfigurable, strain-gauge-based force-sensing sidestick that faithfully emulates the force-based control logic of modern FBW aircraft inside a 6-DoF flight-simulation environment. Across 16 volunteers, the device clearly distinguished proactive (feedforward) from reactive (feedback) control strategies: participants who anticipated and applied continuous micro-adjustments achieved up to an order-of-magnitude lower tracking-error variance and reported lighter workload, regardless of prior flight experience. Strong correlations between objective metrics (error variance, stick-force dynamics) and subjective ratings (Cooper–Harper, NASA-TLX) confirm the simulator’s fidelity in capturing meaningful differences in handling qualities. Objective and subjective data confirmed that effective control strategies led to lower tracking errors and reduced perceived workload. With a total component cost of approximately EUR 108, this work presents a viable alternative to expensive commercial systems, greatly lowering the barrier to entry for high-fidelity simulation research. It provides an accessible and robust platform for academic institutions and training programs to explore advanced control laws, human–machine interaction, and pilot training techniques. The system offers a low-cost, reproducible platform for human-in-the-loop research, supporting advanced studies of flight control behavior, pilot strategy, and workload perception in simulated environments. Future work will integrate low-stroke haptic actuation to provide programmable force-deflection gradients and evaluate multi-axis tasks, further enhancing realism while preserving affordability.

## Figures and Tables

**Table 1:** Key performance characteristics of the force-sensing sidestick.

Parameter	Value	Basis/Source
Rated force range (per axis)	±98.1 N (10 kgf)	Load-cell datasheet (TAL220-10 kg)
Quantisation step (1 LSB)	0.024 N	HX711 gain 128, 24-bit converter
Sampling/reporting rate	80 Hz	HX711 fast mode
Linearity (R^2^)	>0.999	System-level test
Asymmetry Error (max)	<0.1% FS	System-level test
Precision (Worst-case Stability)	<0.05 N (~0.03% FS)	System-level test
Crosstalk (Correlation Coeff.)	*r* < 0.1	System-level test

**Table 2:** Component costs analysis.

Component	Quantity	Unit Cost (EUR)
Base materials and manufacturing	1	50
Strain gauge load cells	4	5
HX711 ADC	2	4
Arduino Micro	1	30
Total cost		108

**Table 3:** Individual participant demographics.

Participant	Age (years)	Flight Hours (h)	Simulator Hours (h)
C1	28	78	500
C2	28	15	100
C3	22	143	40
C4	26	150	40
C5	22	141	40
C6	34	800	57
C7	25	0	30
C8	54	0	26
C9	24	0	1
C10	39	0	6
C11	41	0	10
C12	25	0	1
C13	25	0	1
C14	25	0	1
C15	25	0	2
C16	40	0	20

**Table 4:** Performance metric comparison of best and worst performers.

Metric	Best Performer (C15)	Worst Performer (C11)	Interpretation
Tracking Error Variance (°)^2^	0.96	5.04	Higher precision
Stick Force Extrema Count	295	185	More frequent, fine adjustments
Primary Control Strategy	Feedforward (proactive)	Reactive (error-correction)	Different control paradigms
Total Altitude Gain (m)	~2200	~800	Better energy management
Cooper–Harper Rating	3 (Good)	4 (Fair)	Lower perceived workload
NASA-TLX Effort (%)	50%	95%	Lower perceived effort

## Data Availability

All data are available upon request to the corresponding author.

## Supplementary Materials

The following supporting information can be downloaded at: https://www.mdpi.com/article/10.3390/s25144476/s1, Table S1: Load cell datasheet.

## Abbreviations

The following abbreviations are used in this manuscript:

ADC	Analog-to-Digital Converter
CAD	Computer-Aided Design
CHR	Cooper–Harper Rating
DoF	Degree of Freedom
EADI	Electronic Attitude Director Indicator
FBW	Fly-By-Wire
FS	Full Scale
GPIO	General Purpose Input/Output
HID	Human Interface Device
HITL	Human-in-the-Loop
IQR	Interquartile Range
LSB	Least Significant Bit
NASA-TLX	NASA Task Load Index
PCB	Printed Circuit Board
PGA	Programmable Gain Amplifier
RCAH	Rate-Command/Attitude-Hold
RMS	Root Mean Square
