# Supplementary material for: Low-Cost Strain-Gauge Force-Sensing Sidestick for 6-DoF Flight Simulation: Design and Human-in-the-Loop Evaluation
Source: Sensors (Basel). 2025 Jul 18;25(14):4476. doi: 10.3390/s25144476 (PMC12299703; doi:10.3390/s25144476)
Supplement: Supplementary file 1 [file sensors-25-04476-s001.zip › sensors-3741311-supplementary.pdf]

# Supplementary Material – Table S1

**TAL220-10 kg Single-Point Load Cell (TAL220) – Key Specifications**

| Parameter | Spec. | Unit | Notes |
|---|---|---|---|
| Rated capacity (R.C.) | 10 | kg | 98.1 N full scale |
| Rated output (sensitivity) | 2.0 ± 0.2 | mV $V^{-1}$ | @ 10 V excitation |
| Zero balance | ±0.1 | % FS | |
| Non-linearity | ±0.02 | % FS | project-measured |
| Hysteresis | ±0.05 | % FS | manufacturer |
| Repeatability | ±0.03 | % FS | manufacturer |
| Safe overload | 150 | % FS | |
| Ultimate overload | 200 | % FS | typical |
| Deflection at R.C. | ≤ 0.3 | mm | |
| Input resistance | 1000 ± 15 | Ω | |
| Output resistance | 1000 ± 10 | Ω | |
| Insulation resistance (@ 50 V DC) | ≥ 2000 | MΩ | |
| Excitation voltage (recommended) | 5 – 10 | V DC | 12 V absolute max |
| Operating temperature | –10 … +55 | °C | |
| Compensated temperature | –10 … +40 | °C | |
| Protection class | IP65 | — | sealed gauges |
| Cable | 4-core, 220 mm | — | Ø 0.8 mm PVC |

*Table S1 consolidates the mechanical, electrical and environmental characteristics of the TAL220-10 kg single-point load cell used in the study. Values are manufacturer data unless otherwise noted.*
